# The Political Economy of Health Co-Benefits: Embedding Health in the Climate Change Agenda

**DOI:** 10.3390/ijerph15040674

**Published:** 2018-04-04

**Authors:** Annabelle Workman, Grant Blashki, Kathryn J. Bowen, David J. Karoly, John Wiseman

**Affiliations:** 1 Australian-German Climate and Energy College, The University of Melbourne, Melbourne 3010, Australia; jwiseman@unimelb.edu.au; 2 School of Earth Sciences, The University of Melbourne, Melbourne 3010, Australia; dkaroly@unimelb.edu.au; 3 The Nossal Institute for Global Health, The University of Melbourne, Melbourne 3010, Australia; gblashki@unimelb.edu.au; 4 National Centre for Epidemiology and Population Health, Australian National University, Canberra 0200, Australia; kathryn.bowen@anu.edu.au; 5 Melbourne Sustainable Society Institute, The University of Melbourne, Melbourne 3010, Australia

**Keywords:** health, co-benefits, climate change, political economy

## Abstract

A complex, whole-of-economy issue such as climate change demands an interdisciplinary, multi-sectoral response. However, evidence suggests that human health has remained elusive in its influence on the development of ambitious climate change mitigation policies for many national governments, despite a recognition that the combustion of fossil fuels results in pervasive short- and long-term health consequences. We use insights from literature on the political economy of health and climate change, the science–policy interface and power in policy-making, to identify additional barriers to the meaningful incorporation of health co-benefits into climate change mitigation policy development. Specifically, we identify four key interrelated areas where barriers may exist in relation to health co-benefits: discourse, efficiency, vested interests and structural challenges. With these insights in mind, we argue that the current politico-economic paradigm in which climate change is situated and the processes used to develop climate change mitigation policies do not adequately support accounting for health co-benefits. We present approaches for enhancing the role of health co-benefits in the development of climate change mitigation policies to ensure that health is embedded in the broader climate change agenda.

## 1. Introduction

Anthropogenic climate change remains a pivotal issue on global, national and sub-national scales given the pervasive adverse consequences that are projected. For decades, national and sub-national governments, multinational agencies and inter-governmental entities, non-governmental organizations (NGOs) and scientists have dedicated substantial time and energy to understand its causes and propose effective mitigation and adaptation solutions. The Paris Agreement, negotiated and adopted in December 2015, represents the latest attempt by national governments and others to commit to emissions reduction targets at a global level in order to adequately address the predicted consequences of climate change. At the 21st Conference of the Parties (COP21), 195 Parties to the United Nations Framework Convention on Climate Change (UNFCCC) adopted the Paris Agreement, an unprecedented achievement in the history of international climate change negotiations which has seen COP21 heralded as a success [1]. Despite the elation surrounding COP21, the nationally determined contributions (NDCs) pledged by participating Parties are not commensurate to the catastrophic risks posed by climate change, nor are they sufficiently ambitious given the urgent action required. Projections suggest that current national pledges will result in global average surface temperature warming of approximately 2.7 degrees Celsius (°C) by 2100 [2]. This represents a marked departure from a commitment in Paris to “holding the increase in global average temperature to well below 2 °C above pre-industrial levels and to pursue efforts to limit the temperature increase to 1.5 °C above pre-industrial levels, recognizing that this would significantly reduce the risks and impacts of climate change” [3] (p. 3). Appropriate renewable energy technologies and adequate sources of finance are available; the primary challenge for ambitious climate action remains political will [4]. There is now general political consensus that climate change exists, and that a significant proportion of the action needed to drive rapid economic decarbonization is likely to be undertaken at a sub-national level [5]. However, durable solutions remain evasive and agreement on ambitious action at national and global levels continues at a slow pace given the diversity of considerations and perspectives informing the debate.

The predicted health and other impacts of climate change emphasize that there is both an economic and an ethical imperative for urgent action. From an economic perspective, some systematic economic assessments of climate change have indicated that the benefits of early action outweigh the costs of delayed action on climate change [6,7]. Ethically, there are substantial implications for intra- and inter-generational equity, as climate change will have a severe impact on future generations [8]. With this in mind, climate change and human health researchers contend that consideration of the human health implications of climate change has the potential to enhance climate change action by circumventing the political polarization that can often stifle progress. Research conducted in the United States of America (US) concludes that applying a human health frame to climate change positively influences responses to climate action, irrespective of political persuasion [9,10]. Specifically, health co-benefits present an opportunity to positively inform the development and communication of ambitious climate change policies. The health co-benefits of climate change comprise health benefits that occur indirectly as a result of reductions in the emission of greenhouse gases and other climate altering pollutants [11]. 

Firstly, some health co-benefits have been shown to provide strong and tangible domestic impacts, especially for developing countries, in relatively short time frames [12,13]. Given climate change benefits are often longer-term and diffuse, health co-benefits can attend to “the temporal and geographic mismatch between costs and benefits” [14] (p. 475). Secondly, and on a related note, while uncertainty still exists, some health co-benefit studies can provide a comparatively high level of certainty in the estimated benefits, an unusual advantage of health co-benefits given the uncertainty associated with longer-term estimates relating to climate change [15]. Thirdly, over the past two decades, numerous studies have quantified and monetized local, regional and global health co-benefits of mitigation policies [16]. Notwithstanding the controversy surrounding the monetization approach, these quantifications can be used—and have been by some Parties to the UNFCCC, including the European Union (EU) and the US—to inform the development of mitigation policies, supporting the consideration of health in climate change cost–benefit models [17]. Numerous efforts have been undertaken to strengthen the role of health in the climate change agenda, including enhancing the role of health co-benefits in policy development (see Section 2.2). Despite these efforts, a gap still exists been the potential and actual role of health co-benefits in the development of national climate change mitigation policies. Several explanations have been put forward to explain the lack of political traction of health co-benefits, including a focus on cost minimization and research translation challenges [12,13,18]. 

In this paper, we use perspectives from the: (i) political economy; (ii) science–policy interface; and (iii) power in policy-making literature to support the proposition that additional barriers inhibit the integration of health co-benefits into climate change mitigation policies. Through an examination of the fields of literature indicated above, we identify four key interrelated areas where barriers may exist in relation to health co-benefits: discourse, efficiency, vested interests and structural challenges. As a roadmap for this paper, in Section 2, we summarize the health impacts of climate change and outline the health co-benefits of climate change mitigation, then provide an overview of some of the main efforts to enhance the role of health in the climate agenda. In Section 3, we provide details of the methods used to identify the relevant literature. In Section 4, we stratify our findings from a survey of the literature into the four key interrelated areas. With the theoretical basis established, in Section 5, we use the four key interrelated areas and apply insights extrapolated from the literature to health co-benefits. We then identify strategies that may enhance the consideration of health co-benefits in the development of climate change mitigation policies. The paper culminates with suggestions for further research.

## 2. Background 

### 2.1. The Health Impacts of Climate Change and Health Co-Benefits of Mitigation Measures

In 2009, Costello and colleagues asserted that “climate change is the biggest global health threat of the 21st century” [19] (p. 1693). Climate change is already negatively impacting health, and, if permitted to continue unabated, will exacerbate direct and indirect health impacts to varying degrees across populations [20,21,22,23,24]. Vulnerability to the health impacts of climate change are influenced by various factors including geography, current health status, age, gender, socioeconomic status, and infrastructure that “combine in a complex and place-specific manner” [20] (p. 717). While the attribution of climate change impacts on human health is challenging [25], climate change and health researchers utilize sophisticated scientific methods and long-term datasets, which are increasingly able to quantify and attribute specific health burdens to climate change [26,27]. The IPCC stratifies the health impacts of climate change into one of three classifications: direct impacts, ecosystem-mediated (indirect) impacts, and human institution-mediated impacts [20] (see Table 1).

Understanding the interplay among energy sources, climate change and health is critical to respond to the health impacts of climate change. Accessibility to energy has been fundamental for human development and progress. However, the dominant mode of energy production—the combustion of fossil fuels—has serious ramifications for human health across local, national and global scales [28]. The use of coal, oil and gas for the provision of energy results in the emission of climate-altering pollutants. Longer-term, greenhouse gas (GHG) emissions from the combustion of fossil fuels contribute to climate change, resulting in direct and indirect health impacts as detailed in Table 1. The WHO estimates that climate change will account for 250,000 deaths annually in 2030 given predictions for increasing incidences of malaria, diarrhoea, malnutrition and heat stress [29]. Shorter-term, some emissions affect air quality, which in turn can impact the respiratory and cardiovascular health of populations [30,31,32]. The WHO estimates that in 2012, seven million deaths were attributable to household and ambient air pollution globally [33]. The interrelationship between air quality and climate change is inextricable, with many air pollutants produced concurrently with greenhouse gases through the combustion of fossil fuels [34,35]. Further, climate change exacerbates air quality issues, with projections of increasing premature deaths due to ozone and particulate matter 2.5 micrometers or less in diameter (PM_2.5_) in coming years as a result of climate change [36]. It is unsurprising that there are substantial economic costs attributable to climate change and air pollution. Estimates of the economic costs associated with climate change suggest that global annual gross domestic product (GDP) could be impacted by up to 3.3 percent by 2060; labour productivity constitutes one area that will be most significantly impacted [37]. Further modelling by the Organisation for Economic Cooperation and Development (OECD) estimates that the economic consequences of outdoor air pollution will result in health care costs of US$176 billion and 3.7 billion lost working days annually by 2060 [38].

Given the magnitude of current and projected health impacts, the health community has moved to highlight the potential health co-benefits that result from ambitious mitigation efforts. To determine the potential health co-benefits that arise from domestic and global action, complex modelling techniques have been created and are utilized by researchers, government and non-government organizations [17]. The broad methodological processes underpinning health co-benefits studies have been documented [13,39] and several literature reviews on the co-benefits of climate change mitigation policies have been performed [12,40,41,42]. The findings are consistent; despite the heterogeneity of study methods, prospective health co-benefits studies consistently conclude that the implementation of ambitious mitigation measures can reap significant health benefits for local populations, and partially, if not completely, offset resulting implementation costs [17,43]. A strong appeal of health co-benefits is their immediacy. Specifically, health benefits associated with reduced air pollution can materialise promptly after mitigation measures are implemented [13,44] (see Table 2).

There are some important examples that suggest health co-benefits can and do influence the development of climate change mitigation policies. In China, the adverse health impacts of air pollution are driving emissions reduction efforts [45,46]. In the US, climate change mitigation policies have been pursued through clean air legislation in recent years with health co-benefits publicly communicated as a key selling point [47] in an attempt to pursue climate action despite the politically toxic nature of the climate change debate.

However, there is recognition that co-benefits have not gained commensurate political traction in a majority of Parties to the UNFCCC [12,13,18,48,49]. Limited and varying explanations have been proposed. Some of these explanations align with political economy thinking. For example, Nemet and colleagues (2010) argued that a political “focus on cost minimization—rather than comparison of benefits and costs—diminishes the role of benefits in general” [12] (p. 1). In their literature review of co-benefits, Mayrhofer and Gupta (2016) concluded that, given the dominant influence of economists, the application of “co-benefits ends up being a ‘business-as-usual’ incremental approach which does not adequately call for the structural change needed to address climate change…” [18] (p. 28). Our findings on the role of health co-benefits in the development of Australian climate change mitigation policies support the notion that a number of barriers constrain the consideration of health co-benefits in mitigation policy development, including the disproportionate influence of economic modelling and vested interests [49]. Other conclusions posit alternative explanations. For example, Remais and colleagues (2014) advanced the need to enhance the policy relevance of health co-benefits studies by prompting health co-benefits researchers to “iteratively engage policy makers actively in their work” [13] (p. 453). Others suggest that the science–policy interface presents a number of challenges, and a viable solution requires an integrated approach [50,51]. While the explanations offered to date provide a solid foundation for exploring solutions to enhancing the role of health in the climate change agenda, additional insights may be gleaned from consideration of the political and economic structures and processes that underpin policy development. 

### 2.2. Efforts to Enhance the Role of Health in the Climate Change Agenda

Over the past three decades, extensive efforts have been undertaken to enhance the role of health in the climate change agenda at national and global levels. Firstly, the Intergovernmental Panel on Climate Change’s (IPCC) Working Group II on Impacts, Adaptation and Vulnerability has examined the health impacts of climate change in a standalone chapter since the third assessment report was released in 2001, with a dedicated section on health co-benefits in the latest assessment report released in 2014 [20]. Secondly, in the last decade, the prestigious British medical journal, the *Lancet*, has published several extended series that review the health impacts of climate change [19] and the health co-benefits of mitigation activities [52,53,54,55,56,57]. More recently, the *Lancet* Commission on Climate Change and Health (the Commission) was launched in 2015. Comprising a multidisciplinary consortium of researchers, the Commission provides specific recommendations to government to enhance climate action, and monitors, assesses and reports on progress of health in the climate change agenda [4,58,59]. Thirdly, the health community has an increasingly strong presence in climate change discourse, including at side events that occur concurrently to international negotiations at the COPs [51,60]. Significantly, the “right to health” was explicitly included in the Paris Agreement text. This inclusion constitutes the first time that health has been included in an international climate change instrument since the adoption of the UNFCCC [61]. Finally, the World Health Organization’s (WHO) recently elected Director-General, Dr Tedros Adhanom Ghebreyesus, has indicated that addressing the health impacts of climate change is a priority under his leadership, an issue he reportedly discussed with participants at the Hamburg G20 summit in July 2017 [62]. WHO’s commitment to integrating health into the climate change agenda saw representatives “engaged fully” at COP23 held in 2017 [8] (p. 4).

These efforts are to be commended and have ensured that health has been a consideration, particularly in the development of adaptation planning at the national level. However, it is important to recognize that significant barriers still exist that challenge the consideration of health in the climate change agenda [63]. For example, the WHO acknowledges that the health sector’s access to climate financing remains minimal [8]. Additionally, a 2010 survey of representatives of UN agencies, Parties to the UNFCCC and NGOs found consensus that health has not been of great importance, but should be, in international climate change negotiations and outcomes [64]. Further, in 2016, then Secretary-General of the UNFCCC, Christiana Figueres, addressed the Sixty-ninth World Health Assembly congratulating the public health community for their mobilization in Paris, but also noting that 85 percent of national climate change plans still do not refer to health [65]. The limited traction of health in the climate change agenda is perplexing, given the established biophysical limits to adaptation, as well as the extensive projected costs, including health costs, directly and indirectly associated with climate change [37]. Several solutions to address the discrepancy between the potential and actual traction of health in the climate change agenda have been suggested. These include the need for increased communication, integration, advocacy and leadership efforts by the health community [51,66,67]. However, as Lockwood (2015) rightfully questions, “…if there are so many ‘win-wins’ between emissions reduction, economic growth and improvements in well-being, why haven’t these already been realized?” [68] (p. 149). There is limited research that analyses the bearings of political, economic and policy-making structures and processes on the uptake of health in the climate change agenda. In our view, further interdisciplinary assessment is necessary to better understand why health is, or more importantly is not, considered in high level debates about climate change mitigation.

## 3. Materials and Methods

A literature review was used to identify relevant material for the development of this paper. Until recently, climate change and health research has been relatively sparse. A 2012 inventory of publications indexed in PubMed under “climate change” and “health” identified just over 1500 publications out of almost 20 million total citations [51,69]. Government and non-government agencies have addressed this gap by contributing to the literature with their own research and assessments. Consequently, grey literature, including several government and non-government publications, was identified and included in the paper.

Relevant documents and literature were retrieved between March 2015 and January 2018. To identify relevant peer-reviewed literature, literature searches of three research databases (Web of Science, Scopus, and JSTOR) were performed between May and June 2015. Terms searched included “climate change”, “global warming”, “mitigation policy”, “political economy”, “health”, “health co-benefit”, “public health” and “human health”. Monthly alerts were created for each database to identify additional relevant papers published after June 2015. Additional relevant publications and reports were identified through the review of bibliographies. In total, 1600 documents spanning books, peer-reviewed literature and grey literature were identified. It was not possible to include all documents in this paper. To identify the most relevant documents given the paper’s scope and focus, we initially screened abstracts and executive summaries, searched for key words and phrases, and scanned the documents to familiarize ourselves with the content. This rapid thematic analysis enabled us to establish the four key interrelated areas and identify the final documents that were included in this paper.

## 4. Results

A review of the literature on the political economy of health and climate change, the science–policy interface and power in the policy-making process facilitated the identification of four key interrelated areas where barriers may exist for health co-benefits: discourse, efficiency, vested interests and structural challenges. The literature in these fields is expansive and contested, and the overview presented below is by no means exhaustive. We highlight some of the central tenets and examples from these fields to guide a meaningful discussion on the implications for health co-benefits and to aid the consideration of strategies to enhance their uptake in the development of national climate change mitigation policies. 

### 4.1. Discourse

The political economy literature portrays the dominant discourses for both climate change and health as unduly influenced by economic forces. In relation to climate change, for example, at both national and global levels, climate change discourse is embedded in an economic frame, where the problems and the primary solutions are economic in nature. Climate change can be viewed as an economic problem given failures of the market to internalize the costs of using certain environmental “goods” [70]. In relation to solutions, it has been asserted that policy-makers have relied heavily upon economic approaches to solve climate change by focusing on efforts to internalize externalities via market-based interventions such as pricing mechanisms, including emissions trading schemes, cap-and-trade systems and carbon taxes. In this way, “climate change action has been transformed, largely through the agency of the state, into the generation of tradable, priced and ownable units of molecular ‘mitigation’” [71] (p. 481). In pursuing an economic solution, there are claims that governments perpetuate neoclassical economic practices [72]. 

In relation to health, some political economists argue that in societies where profit primarily motivates economic and social decision-making, health is defined in a functional manner. This perspective critiques the capitalist system by presenting it as reducing an asset-less individual’s value to their capacity to generate productivity through labour. In other words, health is inextricably linked with an individual’s value to society; sickness is symbolic of “…an inability to produce profit…” [73] (p. 8). Further, within this profit-driven structure, a political focus on short-term, quantifiable outcomes poses “particular problems for public health, which is, by its very essence, concerned with long-term outcomes” [74] (p. 95). The neoliberal pursuit of individualism, which “holds individuals totally responsible for their actions and the consequences, including health” [74] (p. 74), has serious implications for health outcomes. By focusing on individual experiences of illness, political, economic, social and environmental factors that may contribute to ill health are easily overlooked [75]. An individualistic approach to health encourages victim blaming [74], which oversimplifies the often complex and convoluted nature of illness, ignoring “the social, cultural and economic context in which decisions are taken” [74] (p. 80).

### 4.2. Efficiency

As an extension of the economic dominance of climate change and health discourse, and in line with mainstream economic principles regarding resource allocation, the political economy literature tends to argue that economic efficiency is central to climate change policy decisions. Climate change negotiations are preoccupied with questions surrounding the economic optimization of emissions reduction and the distribution of responsibility in achieving this outcome [76] (p. 915). There is recognition that, to determine “optimal” carbon reduction commitments, that is the most efficient policy option, economic instruments laden with neoclassical assumptions are regularly used as the basis for determining policy options. These instruments, often integrated assessment models (IAM) such cost–benefit analysis (CBA) and computable general equilibrium (CGE) models, are used to determine policies that are politically and economically pragmatic as opposed to optimal for environmental and social outcomes [77] by mixing “descriptive analysis and value judgements in ways that deserve close and critical scrutiny” [78] (p. 299). The assumptions embedded into these instruments critically inform the final policy outcome, and are regularly contested (e.g., [79]). While it is not feasible here to review all of the contestations around modelling, two notable examples are worth highlighting. The first example relates to the “optimal” discount rate to apply to future benefits and costs. A discount rate is used to account for the discrepancy between current and future costs and benefits, by reducing the current value of a cost or benefit that will not be realized for a period of time [80]. Put differently, a discount rate “implies that the well-being of this generation matters more than that of its children, who in turn matter more than their children” [78] (pp. 300–301). The discount rate used significantly influences the economic output that ultimately informs the preferred policy outcome [81], with far-reaching consequences for inter-generational equity. For this reason, the 2006 Stern Review—one of the most comprehensive and longer-term economic cost–benefit analyses of climate change—used a comparatively low average discount rate [6], a decision that was criticized by a number of economists [82].

A second example relates to the technically and ethically complex processes used to assign monetary values to “invaluable assets”, such as human life and health. Currently, different models use different estimates to determine the valuation of a human life, such as an individual’s willingness to pay, or national income levels. This has resulted in some economic assessments valuing the lives of individuals in richer countries more than the lives of individuals in poorer countries [78]. Such approaches reaffirm that intra-generational inequity remains a key issue in the development of climate change policies.

### 4.3. Vested Interests

An overlapping area covered in the political economy and power in policy-making literature is the role of vested interests in influencing policy outcomes. Despite repeated efforts to demonstrate the economic efficiency of implementing climate change policies, “in reality governments inevitably get it wrong, in part because they are in hock to vested interests” [68] (p. 149). Some political economists explain this as partially the result of another relevant yet contested principle underlying economic considerations in the development of policies: the Pareto Principle. The principle holds that any economic change or redistribution is permissible only if the situation improves for one or more individuals without negatively impacting the situation of another. With the reality that almost every change results in winners and losers, the consideration of this principle often reasserts a neoclassical perspective that non-intervention in the market is the optimal response [83]. There is a cognizance of the importance of who wins and who loses in climate change action. For example, in his thesis on the political economy of the environment, Boyce (2002) hypothesizes that the power dynamic between the winners—that is, those who experience net benefits from an activity—and the losers, or those who endure net costs, directly influences the level of environmental degradation that ensues; environmental degradation is greater if the winners are relatively powerful compared to the losers [84]. This hypothesis again highlights issues of intra- and inter-generational equity. Boyce’s hypothesis is confirmed by Steves and Teytelboym (2013), whose comparative assessment of the climate change mitigation policies of 95 countries concluded that the size of a country’s carbon-intensive industry was a major factor influencing climate change policy adoption [85]. This has led some political economists to support the position that “…the capitalist market economy is the problem, not the solution. In its modern form, shaped by corporate power, consumerist practices, and the prevailing ethos of individualism, it stands as the antithesis of ecological sustainability” [70] (p. 334). Similarly, with health, there is recognition that powerful vested interests exist that can and have influenced public health interventions. Tobacco control policy represents one example where vested interests have been implicated in delaying meaningful policy development. Consequently, parallels between tobacco control and climate change policies have been drawn by climate change and health researchers [86].

The role of power and vested interests in policy-making is further elucidated when examining key theoretical explanations of the policy-making process. Theoretical positions on the policy-making process exist on a continuum. At one end exist idealistic theories, such as the “rational actor model” that presents policy-making as a logical, linear and tidy process involving a comprehensive assessment of all information in order to produce an optimal policy outcome [87]. The middle ground is populated by theories such as incrementalism and bounded rationality, which suggest the policy-making process is more opportunistic and iterative in nature, occurring within pragmatic parameters, such as time, information and individual abilities [87]. At the other end, theories such as Cohen and colleagues’ (1972) “garbage can model” and Kingdon’s (1984, 1995, 2011) “multiple streams” approach to policy-making are found. These understandings of the policy-making process conceptualize a messy combination of problems, solutions and participants that interact in a non-linear and almost serendipitous manner to produce policy outcomes. The latter theories support the notion that many actors are involved in the development of policy, none of whom can be considered to hold neutral positions [88].

While governments are ultimately responsible for making policies, it is well understood that the various actors contributing to the policy-making process use resources at their disposal in an attempt to influence the final policy outcome. In this way, “governments set the field of play and the rules for debate … but energy comes from actors on the field” [89] (p. 1086). As such, the concept of power is central to the policy-making process [90]. Accordingly, there are additional theoretical perspectives on who wields power in the policy-making process. Dahl’s (1961) pluralist perspective, considered the dominant theory in liberal democracies, understands power to be distributed among individuals and groups within society. The role of the state is to act as adjudicator in managing competing interests inevitable in the policy development process [90]. Public choice theory extends this understanding of power in the policy-making process by recognizing the state as an interest group, with elected officials and bureaucrats pursuing their own self-interests, resulting in distorted policies that benefit certain groups at the expense of the public interest [90]. Critiques of the pluralist perspective, most notably put forward by Bachrach and Baratz (1962), assert that power is not always overt; “much power is exercised more covertly and through subtle cultural processes…” [91] (p. 31). Bachrach and Baratz coined the term non-decision making to capture this concept, arguing that power “often is exercised by confining the scope of decision-making to relatively ‘safe’ issues” [92] (p. 948). These analyses of the policy-making process illuminate the ways in which many actors, including experts and those with vested interests, can inform the final policy outcome.

### 4.4. Structural Challenges

The literature identifies that several structural and procedural challenges exist in relation to health and climate change. Firstly, in relation to health, there is an acknowledgement that complex and dynamic interactions between various domains—political, economic, social, and cultural—inform an individual’s or a population’s health status [93]. These determinants of health often fall outside of what is generally considered the realms of the health sector [94]. As a result, the health sector is limited in its capacity to address in totality many health issues experienced. In relation to climate change, a “web of stakeholders” are relevant to climate change policy decisions [95] (p. 1034). Included in the list of actors often recognized as influencing the policy-making process are scientists or experts, who legitimize the process by providing objective, evidence-based inputs [96]. Researchers have considered the ways in which scientific knowledge can be used in the policy-making process. Schrefler (2014) proposed that the use of expert knowledge in regulatory policies falls into one of three categories: instrumental, strategic or symbolic. The *instrumental* use of experts sees them engaged in the policy process to determine the best solution to a problem. The *strategic* use of experts sees them involved in the policy process to support a pre-defined policy position. The *symbolic* use of experts sees them contribute in order to strengthen the legitimacy of the policy makers [96]. Schrefler outlines a number of potential explanations for the exclusion of expert knowledge in the policy-making process, including that “pre-existing approaches to tackle and decide on a given policy issue are so entrenched … that expertise does not really make a difference when decisions are taken, particularly when these decisions trigger only small incremental changes in existing policies” [96] (p. 71). While scientific evidence does not always gain the political traction it warrants, there are opportunities to enhance the role of scientific knowledge in the policy-making process.

Cáceres and colleagues (2016) provided insight gained from their research in Argentina. The “science deficit model” maintains that low uptake of scientific research in policy development can be explained by poor communication of scientific findings by scientists to policy-makers, or the inability of policy-makers to interpret scientific findings appropriately; “it is basically a technical-communicational problem” [97] (p. 57). Cáceres et al. determined that this theory is problematic in its oversimplicity of the policy-making process. They identified the “power dynamics model” as more representative; this conceptualization of the policy-making process recognizes that while pivotal, science knowledge represents just one element in a “highly contested, non-linear and multi-sectoral field where institutions, subjectivities, values, interests, power relationships as well as knowledge, play a role” [97] (p. 62). Based on their experiences, the authors offered four considerations for enhancing the role of science in the policy-making process. Scientific knowledge is most likely to be incorporated when: (i) “it aligns with the interests of sectors that concentrate the larger shares of political power in society”; (ii) it is “encapsulated in compelling, widely-communicated storylines…well understood and appropriated by society”; (iii) “it has been appropriated by, and is well integrated into the agenda of a wide range of social actors with active representation in the negotiation process”; and (iv) it can “contribute to create or take advantage of social-political windows of opportunity” [97] (p. 63). 

These considerations are pertinent when searching for additional explanations to understand the undervalued role of health in the climate change agenda. We now turn our attention to considering how the four key interrelated areas explored above can be extrapolated to provide a better understanding of the barriers and opportunities for health co-benefits in the development of climate change mitigation policies.

## 5. Discussion

Applying insights from the literature on the political economy of health and climate change, the science–policy interface, and power in the policy-making process supports the proposition that current political and economic structures and processes create several barriers for the inclusion of health co-benefits in the development of climate change mitigation policies. It is imperative that researchers are aware of the implications of these challenges; if researchers wish to enhance the political traction of health co-benefits, understanding the complex politico-economic paradigm is vital. Using the four interrelated key areas identified in Section 4, we transpose insights from the literature to illuminate additional barriers for health co-benefits in the development of climate change mitigation policies.

### 5.1. Discourse

In a globalized, market-oriented environment, the dominant climate change discourse has focused on the shorter-term costs of action and on “fair” calculations of burden sharing at the expense of meaningfully incorporating the costs of inaction into the policy-making process. This global framing permeates national levels of policy-making, where “economic growth remains so central to political legitimacy” [68] (p. 149). Consequently, many national governments are beholden to the supremacy of economic guidance that focuses on least-cost pathways and the identification of the most “efficient” policy options. Further, in a policy-making environment where the benefits can be difficult (but not impossible) to quantify, it is simpler for policy-makers to disregard the qualitative evidence than to try and justify its inclusion in the policy development process. In Australia, a lack of domestic quantitative health co-benefits assessments has undermined the role of health co-benefits in the development of climate change mitigation policies [49]. These barriers are exacerbated by the realities of perceptions around health; with individuals considered ultimately responsible for their health, attributing and communicating the health impacts of shorter- and longer-term climate change becomes increasingly challenging. The dominant discourse is further compounded by the reality that the policy agendas of health ministers are often “crowded with many demands, and influenced by competing and conflicting interest groups” [98] (p. 142). Such political realities support the de-prioritization of health co-benefits in the development of climate change mitigation policies.

### 5.2. Efficiency

For many governments, a focus on optimizing cost-effectiveness results in policy-makers pursuing health gains through direct policies. A notable example is in relation to air quality, where it is cheaper to implement measures to reduce local air pollution than to address air quality through climate policies [12]. An exception to this view, as mentioned above, relates to the Obama Administration’s pursuit of climate change mitigation policies through air quality legislation in 2015 to avoid a politically hostile Congress in the lead-up to COP21. In the EU, however, health remains a primary justification for the pursuit of stringent clean air standards [99]. While health co-benefits are accounted for in the development of climate change mitigation policies in the EU through the use of IAMs, they do not significantly inform the final policy outcome. In Australia, health co-benefits have only been considered meaningfully in the development of national vehicle emissions standards [49]. The influence of health outcomes as a clearly defined justification for air quality policies is noteworthy, yet this can undermine the consideration of health co-benefits in the development of climate change mitigation policies. This is especially problematic given there can be substantial trade-offs between isolated policy goals of reducing air pollution and abating climate change [100,101,102].

### 5.3. Vested Interests

The structure of the Paris Agreement, which requires each Party to the UNFCCC to make regular emissions reduction pledges of increasing ambition, will create economic conflicts of interest between stakeholders and sectors domestically [103]. The implementation of mitigation policies, often regulatory in nature, naturally creates winners and losers [96]. Longer-term, winners of ambitious climate change mitigation policies comprise nearly all sectors, including the renewable energy sector, as well as current and future populations, with benefits diffuse across space and time, and difficult to measure. Losers are likely to be big corporations as well as extractive industries, often able to exert undue power and influence over the policy-making process [103]. In Australia, for example, there is acknowledgement that business and industry stakeholders are highly influential in the climate change policy-making process as a result of corporate contributions to economic growth and stability [49]. Conversely, those who suffer most from a delay in effective climate change policies are the most vulnerable populations with minimal to no power in the policy-making process: children, economically disadvantaged populations and future generations. Vested interests are not limited to corporate interests eager to maintain the status quo. Outside of the more obvious economic motivations that exist for the extractive industry, strong ideological motivations have been linked to the climate change denial movement that has particularly strong roots in the US. Specifically, “a staunch commitment to free markets and disdain of governmental regulations” remains a defining feature of climate change denialists, who appear determined to uphold the “modern Western social order” that is often characterized by political and economic conservatism [104] (p. 144). This perspective is supported by analysis performed by Jacques and colleagues (2008), which confirmed a strong link between environmentally sceptic publications and conservative think tanks [105].

### 5.4. Structural Challenges

While an integrated approach is optimal for the development of a cross-sectoral issue such as climate change, the politico-economic realities limit this approach in practice. Different approaches to problems, the use of diverse technical language, and the political reality of bounded rationality complicates cross-sectoral integration efforts. In federated systems, integration challenges are exacerbated given “the potential for differences of ideology and political interests between levels of government…have provided fertile ground for blame-shifting and regulatory complexity” [98] (p. 139). Further, environmental health concerns have tended to be addressed by proposals from environmental agencies, departments and NGOs, as opposed to health departments. In the US, for example, the US Environmental Protection Agency (EPA) was responsible for developing the *Clean Power Plan* and disseminating the health co-benefits [47]. Similarly, in the EU, the Directorate-General for Climate Action and the Directorate-General for Environment coordinate the development of mitigation policies, with the quantification of health co-benefits supported by the European Commission’s Joint Research Centre and other external research institutes, such as the International Institute for Applied Systems Analysis. While national health ministries may logically be considered best placed to provide in-house expertise and to advocate for health in the climate change agenda, often acute health care concerns are dominant for health ministries [98,106]. Political short-termism and a pragmatic governance style can also undermine optimal policy outcomes, particularly for a cross-sectoral, longstanding policy area such as climate change. For example, analysis of the United Kingdom’s climate policy development by Gillard (2016, 2017) determined that, in the aftermath of the global financial crisis in 2008, climate change policy was considered too expensive to pursue and austerity measures inevitably de-prioritized the implementation of ambitious climate action [107,108].

### 5.5. Strategies for Enhancing Health Co-Benefits in Climate Change Mitigation Policies

The identification of additional barriers for health co-benefits based on insights from the literature helps to further elucidate the lack of traction they have garnered in policy development to date. These barriers are substantial, and have led some researchers to conclude that “the immediate *perceived* costs and political barriers (in spite of net co-benefits) are likely to remain substantial, until serious impacts of warming become so obvious after a dangerously long period of further business as usual, that public perceptions change and political resistance also collapses” [109] (p. 188). However, armed with the knowledge that the policy-making process is regularly “ruled by dominant narratives, economic and political structures, or by the interests of the most powerful players” [97] (p. 63) provides opportunities to explore strategies to exploit the political and economic structures and policy-making processes that undermine the role of sectors such as health in the climate change agenda. Returning to Cáceres and colleagues’ four considerations for enhancing the role of scientific knowledge in the policy-making process, we propose four strategies that may enhance the relevance and influence of health co-benefits in the development of national climate change mitigation policies. 

First, aligning the health co-benefits of mitigation with the pursuit of national renewable energy goals is essential. Signatories to the Paris Agreement have in principle committed to transitioning to low-carbon economies [3]. As identified in Section 5.3, the climate change action associated with this shift will inevitably produce winners and losers. With energy security of paramount importance to many national governments [110], emphasizing the dual benefits to energy security and health that result from ambitious climate change action may appeal to policy-makers. In their multi-model analysis of the co-benefits of a suite of mitigation measures in the EU, Scwanitz and colleagues (2015) conclude that the immediate implementation of mitigation measures would see the EU reduce its dependence on imported fossil fuels, thereby enhancing energy security in Europe [111]. There is an opportunity for an increasingly powerful alliance to be built between winners of climate change action—such as the health sector and the renewable energy sector—to destabilize the undue influence of the extractive industries in the policy-making process.

Second, and closely related to the first strategy, strengthening the position of health in the climate policy community through the identification of several influential champions for health would greatly assist in embedding health in the climate change agenda. Positioned across the private and public sectors, such champions may not necessarily sit within the health sector but they would need to be “at the table” or consulted during climate change mitigation policy development. 

Third, developing and maintaining a compelling narrative with several threads that resonate with diverse groups within the community is necessary. The first, and arguably the most important, narrative must directly challenge the misconception that climate action is primarily a burden by firmly shifting attention to the many benefits, including those relating to health, that result from climate action. Given the dominance of neoclassical economic thinking, highlighting the positive implications for businesses that arise from climate action may enhance traction. The US EPA estimated the labour productivity gains that would result from the implementation of the *Clean Power Plan*—300,000 fewer missed work days—and used this statistic as one of five key selling points for the emissions reduction initiative [47]. 

Finally, continuing to utilize opportune occasions to communicate the health consequences of climate change, and the health co-benefits that result from strong climate action, to both the politicians and the community is pivotal if the role of health co-benefits in mitigation policy development is to be enhanced. The WHO and others continue to estimate morbidity and mortality rates attributable to environmental risk factors, including climate change (e.g., [112,113]). Ensuring that robust, timely evidence is accessible for champions and other knowledge brokers at times when climate change is thrust back to the top of the political agenda will maximize the prospect of firmly embedding health in the climate change agenda. 

## 6. Conclusions

Current efforts to address climate change are inadequate given the projected health and other impacts. Consideration of health, specifically health co-benefits, has been recognized by climate change and health researchers as a strong strategy to encourage more ambitious climate change action. However, evidence suggests that health co-benefits have not gained the political traction they warrant. While several barriers have been identified, we applied insights from literature on the political economy of health and climate change, the science–policy interface and power in the policy-making process to identify additional barriers for health co-benefits. This approach provides a unique perspective on the challenges of meaningfully incorporating health into the climate change agenda. Based on the literature, we identified four key interrelated areas where barriers are likely to exist and inhibit the role of health co-benefits in the development of climate change mitigation policies. Based on insights in these areas, we proposed implications for health co-benefits and provided potential strategies that may assist in enhancing the uptake of health co-benefits in the development of climate change mitigation policies.

Our review of the literature identified current gaps in research that would help to strengthen an understanding of the barriers inhibiting the role of health co-benefits. Firstly, we have been unable to locate research that specifically analyses the power dynamics between national environment, energy and health ministries. A better understanding of the interactions among these ministries may provide further clarity and insights for enhancing the role of health co-benefits in the development of national climate change mitigation policies. Secondly, as previously mentioned, there is limited research that investigates the role of health co-benefits in the development of national climate change mitigation policies for individual Parties to the UNFCCC. This research is imperative for reinforcing our current understanding of the barriers impacting the traction of health co-benefits in the development of climate change mitigation policies, and health in the climate change agenda more broadly.

## Figures and Tables

**Table 1:** An overview of the health impacts of climate change. Adapted from IPCC AR5 (2014) [20].

Classification	Potential Impacts
Direct	Increased mortality resulting from increased exposure to: hot and cold weather extremes; floods and storms; ultraviolet radiation.
Ecosystem-mediated	Increased morbidity and mortality from increased exposure to: vector-borne and other infectious diseases; food- and water-borne infections; air pollution and aeroallergens.
Human institution-mediated	Increased morbidity and mortality from poor nutrition; occupational health; mental health; violence and conflict.

**Table 2:** Examples of potential health co-benefits from mitigation activities relating to the energy and transport sectors, including the anticipated time lag for the realization of health co-benefits. Adapted from Remais et al. (2014) [13].

Mitigation Activity	Potential Health Co-Benefit(s)	Anticipated Time Lag(s)
Reductions in fossil fuel use	Reductions in sudden cardiac death risk; acute respiratory infections; chronic obstructive pulmonary disease exacerbations	Days to weeks; weeks and months; weeks and months
Improvements in fuel economy; incentivize electric vehicle use; tighten vehicle emission standards		
Increases in accessibility to active modes of transport, including walking and cycling	Reductions in type 2 diabetes; depression; breast and colon cancer incidence	Years for all potential health co-benefits identified
